# Dexmedetomidine combined with ropivacaine for erector spinae plane block after posterior lumbar spine surgery: a randomized controlled trial

**DOI:** 10.1186/s12891-022-05198-9

**Published:** 2022-03-11

**Authors:** Wang Yi-han, Tang Rong, Li Jun, Wang Min, Zhang Yan, Li Yi, Liu Jie-ting, Huang Sheng-hui

**Affiliations:** Department of Anesthesiology, Lanzhou University Second Hospital, Lanzhou, 730000 People’s Republic of China

**Keywords:** Dexmedetomidine, Ropivacaine, Erector spinae plane block, Posterior lumbar spine surgery, Postoperative analgesia

## Abstract

**Purpose:**

Due to lumbar spinal surgery is frequently accompanied with moderate-to-severe postoperative pain, it is necessary to find an effective postoperative analgesia for patients with this surgery. This study aimed to observe the analgesic effect of dexmedetomidine combined with ropivacaine erector spinae plane block (ESPB) used in posterior lumbar spine surgery.

**Methods:**

In this clinical trial, patients undergoing posterior lumbar spine surgery were recruited and randomly divided into two groups: intervention and control. The intervention group (Group E) received 0.375% ropivacaine with 1 µg/kg dexmedetomidine in a total of 20 ml for ESPB; the control group (Group C) received 20 ml ropivacaine 0.375% for ESPB. US-guided ESPB was performed preoperatively in all patients. Demographics, anesthesia time, surgery time, and ASA grade from the participants were recorded at baseline. The primary clinical outcome measures were 2-, 4-, 8-, 12-, 24-and 48-h visual analog scale (VAS) pain scores after surgery at rest and movement state. Other end points included opioid consumption, number of PCIA presses, flurbiprofen-axetil consumption, quality of recovery and pain management after surgery.

**Results:**

One hundred twenty patients were enrolled in the study (mean [SD] ages: Group E, 54.77 [8.61] years old; Group C,56.40 [7.87] years old; *P* = 0.280). The mean anesthesia time was 152.55 (15.37) min in Group E and 152.60 (16.47) min in Group C (*P* = 0.986). Additionally, the surgery time was 141.70 (15.71) min in Group E compared to 141.48 (17.13) min in Group C (*P* = 0.943). In addition, we found that the VAS pain scores in the resting state during the postoperative period at 8–48 h were lower in Group E than in Group C. However, the VAS pain scores in the active state were lower in Group E at 12–48 h (*P* < 0.05). More importantly, the consumption of opioids and flurbiprofen-axetil after surgery was also lower in Group E (*P* < 0.05). Subsequently, we administered questionnaires on the quality of recovery and pain management after surgery that were positively correlated with the postoperative analgesic effect. It was worth affirming that the QoR-15 scores and APS-POQ-R questionnaire results were different between the two groups, further confirming that the combination of drugs not only could obtain an ideal analgesic effect but also had no obvious adverse reactions (*P* < 0.05).

**Conclusions:**

All the findings suggested that dexmedetomidine could significantly relieve postoperative pain and reduce the consumption of opioids in patients undergoing posterior lumbar spine surgery without obvious adverse reactions as a local anesthetic adjuvant. Further studies with larger sample sizes and different drug dosages may be useful in understanding the potential clinical benefits of dexmedetomidine.

## Introduction

Enhanced recovery after surgery (ERAS) is a multimodal, multidisciplinary approach to promote postoperative outcomes by applying multiple evidenced-based interventions, which have been recently adapted for spine surgery at multiple institutions worldwide. While the role of ERAS protocols has been demonstrated in spinal surgery, the area of perioperative pain management requires more dramatic and complete improvements so that patients can benefit from implementing ERAS protocols after surgery [1].

The key strategy to enhance perioperative pain management includes the use of a multimodal analgesia approach to reduce opioid consumption, decrease time in the hospital and improve patient satisfaction [2]. Ultrasound-guided nerve block has become a crucial component of the multimodal analgesia approach [3]. A large number of studies have shown that the perioperative analgesic effect of nerve block is superior to that of other drugs [4, 5]. Therefore, it is necessary to implement nerve block for posterior lumbar spinae surgery.

ESPB is a paraspinal interfascial plane block that targets the ventral and dorsal branches of the spinal nerve. Local anesthesia is injected between the deep fascia of the erector spinae muscle and the vertebral transverse process during ultrasound-guided block [6]. Several studies [7–11] have demonstrated that the demand for opioids in the ESPB group was significantly lower than that in the control group, and the postoperative satisfaction of the patients was higher after thoracic and abdominal surgery. Additionally, clinical case reports have reported the use of ESPB leading to effective postoperative analgesia management in spinal surgeries [12, 13]. Therefore, ultrasound-guided ESPB could make an important contribution to the management of postoperative pain after spinal surgery [14]. However, the duration of postoperative analgesia can only be maintained for 6–8 h even when medium- and long-acting local anesthetics are used [15]. The potential for prolonging the duration of analgesia after single-injection ESPB is especially important.

Dexmedetomidine is identified as a highly selective short-acting alpha-2 agonist with sedative, anti-anxiety, inhibition of perioperative sympathetic excitation and hypnotic effects [16]. Another potential use of dexmedetomidine in the perioperative management of postoperative pain is as an adjunct to regional anesthesia [17, 18]. For example, in a meta-analysis of more than 2,000 patients, the addition of dexmedetomidine to brachial plexus blocks led to faster block onset, longer duration of blocks, improved analgesia and a significant reduction in morphine consumption [19]. Notably, there have been multiple studies claiming that dexmedetomidine-assisted local anesthetic agents for ESPB extended the duration of block in patients undergoing modified radical mastectomy and curative-intent open thoracotomy [15, 20]. However, whether interfasical dexmedetomidine prolongs the duration of single-injection ESPB and reduces postoperative opioid consumption after open posterior lumbar spinal fusion surgery remains uncertain. Thus, the purpose of this study was to explore the use of dexmedetomidine in ESPB for posterior lumbar spine surgery to better understand this approach.

## Methods

### Study participants

This study was approved by the Ethics Committee of Lanzhou University Second Hospital, Lanzhou, Gansu Province on 08/09/2020 (2020A-043), and the study was registered in the Chinese Clinical Trial Registry (ChiCTR2000038037). All of the enrolled patients had to sign a written informed consent form for this trial before surgery. A total of 120 patients aged 18–70 years old with ASA I–III who were scheduled for 1- or 2-level open posterior lumbar spinal fusion surgery under general anesthesia between January and June 2021 were enrolled in this study.

The exclusion criteria included puncture site infection, abnormal blood clotting function, local anesthetic drug allergy, severe heart and lung disease, arrhythmia, liver and kidney insufficiency or mental illness, a history of chronic pain or a long-term history of taking analgesics. The rejection criteria included severe complications or accidents during the perioperative period or anesthesia, and those who did not cooperate well with the VAS scores.

### Random selection of patients

The included patients were randomly divided into two groups at a ratio of 1:1 using a computer-generated random number table. The results of the distribution were sealed in an opaque envelope and kept by the research administrator. On the day of the operation, the main researcher handed the envelope to the assistant anesthesiologist for the preparation of the test liquid.

### Study procedure

After all of the patients entered the operating room, pulse oxygen saturation, noninvasive arterial blood pressure, and electrocardiograms were routinely monitored. The patients were placed in the prone position and received midazolam 3 mg intravenously for preoperative sedation. The ultrasound probe was placed on the sagittal axis of the horizontal midline of the L3 vertebral body. The spinous processes were first observed, and then the probe was moved laterally to observe the transverse process and erector spinae muscle approximately 3 cm from the midline. Local anesthesia was injected between the deep fascia of the erector spinae muscle and the vertebral transverse process during ultrasound-guided block. An ultrasound-visible puncture needle was inserted from the cranial portion to the caudal portion using the in-plane technique, and the correct position of the needle was confirmed by injecting 2–3 ml of saline solution, after which 20 ml of 0.375% ropivacaine was administered. The same procedure was also performed on the opposite side. We performed injection at the L3 level because ESPB at this level can subsequently spread to levels L1-L5 [14], any of which were potentially affected in our patient sample. In contrast, lower thoracic injection of ESPB could spread only to levels L2-L3 [21]. Sensory examination was conducted with a hot–cold test 30 min after the procedure. Blockade was considered successful only if anesthesia was present in the L1-5 cutaneous area during the sensory examination. In the intervention group, the procedures were the same except that 1 µg/kg dexmedetomidine was added to ropivacaine. After injection, the patient's heart rate (HR) and blood pressure were observed. If bradycardia or hypotension occurred, timely treatment was required.

The patients were given full preoxygenation and induction of anesthesia was performed with intravenous 2 mg/kg propofol, 0.5 ug/kg sufentanil and 0.2 mg/kg cis-atracurium. The maintenance of anesthesia was established with 4–6 mg/kg/h propofol and 0.15–0.3 µg/kg/min remifentanil continuous pumping to maintain BIS values between 40–60. When necessary, 0.1 mg/kg cis-atracurium could be given for muscle relaxation during the operation. The drug continuous infusion dosage was adjusted to maintain HR and mean arterial blood pressure (MAP) within 80–120% of baseline. Hypotension (MAP < 80% of baseline) lasting for 3 min was managed with a bolus of 6 mg ephedrine. Bradycardia (HR < 45/min) was treated with atropine (0.25–0.5 mg).

More importantly, posterior lumbar spine surgery was performed on all of the patients by the same surgical team and using the same techniques. Immediately after surgery, all of the patients were treated with 1 mg of neostigmine and 0.5 mg of an atropine antagonist muscle relaxant, and transported to the postanesthesia care unit (PACU) with tracheal intubation until tracheal extubation indications were reached.

### Postoperative management

The same postoperative management protocol was adopted in both groups. First, patient-controlled intravenous analgesia (PCIA) was administered in the PACU by connecting an electronic infusion pump that consisted of hydrogen morphine ketone with 16 mg of ondansetron. The concentration of hydrogen morphine ketone was set to 0.05 mg/ml, the loading dose was 0.5 mg, the lockout interval was 15 min, and a 0.05 mg bolus was maintained for 48 h. Second, all of the patients received 50 mg of flurbiprofen-axetil intravenously after surgery, which could be repeated when the VAS score was greater than 5 postoperatively. Third, the patients with no apparent discomfort and modified Aldrete scores of 9 or more could be transferred to the ward. Finally, the patients were encouraged to perform functional exercises of straight leg elevation of the lower limbs after being fully awake. Lumbar and back muscle functional exercises were started 48 h after the surgery. On the fourth day, the patients were encouraged to move properly with the protection of a chest and waist brace.

### Outcome measures

Postoperative care and evaluations were performed by a researcher who was unaware of the study groups. The primary outcomes were 2-, 4-, 8-,12-, 24- and 48-h VAS pain scores at rest and movement state after surgery (VAS = 0, no pain; VAS = 10, the most severe pain). The movement state was defined as moving from the supine position to semiseated position. Secondary outcomes included opioid consumption, PCIA press times and flurbiprofen-axetil consumption. In addition, some questionnaires on the quality of recovery and pain management that were positively correlated with postoperative analgesic effect were administered after surgery. Patients were questioned at 24 h, 48 h, and 1 week postoperatively using the 15-item Patient-related Quality of Recovery Questionnaire (QoR-15). The quality of pain management was assessed using the revised American Pain Society Patient Outcome Questionnaire (APS-POQ-R).

### Statistical analysis

To determine the minimal sample size for the primary outcome, we treated the VAS score as a continuous variable and hypothesized a significant difference of 1 in the VAS score at rest at 12 h after surgery. Our pilot study showed a mean VAS score of 4.5 ± 0.8 at rest at 12 h after surgery for the control group and 3.4 ± 1.1 for the intervention group. Student’s *t* test was selected, and the group allocation ratio was equal. Thus, we calculated that a sample of 48 patients would provide 96% power at a 2-sided α level of 0.05. Ultimately, we recruited 60 patients in each group for a total of 120 patients considering possible dropouts and incomplete follow-up.

Statistical analysis was performed using SPSS software, version 23.0 (SPSS Inc. Chicago, USA). The distribution of variables was evaluated for normality using the Kolmogorov-Smirnovand test and histogram test. Normally distributed variables are reported as the mean ± standard deviation (SD) and were analyzed using the independent samples *t* test. Nonnormally distributed variables are presented as the median ± quartiles (IQR) and were analyzed using the Mann–Whitney* U* test. Categorical variables are presented as numbers (percentages) and were analyzed by using the χ2 test. We considered *P* < 0.05 (two-tailed) statistically significant.

## Results

**Fig. 1:**
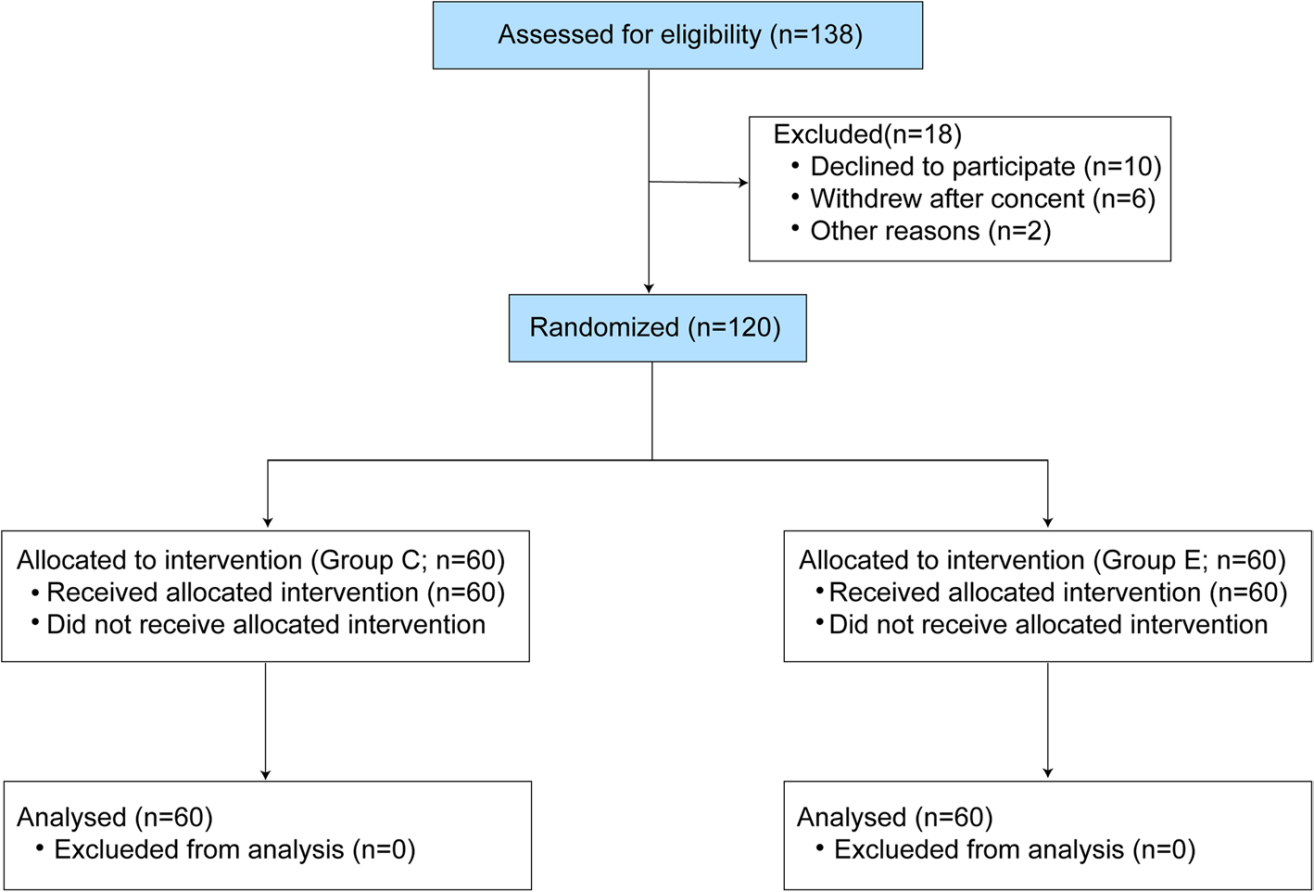
Flow diagram of study

Of the 138 patients assessed for eligibility, ten patients declined to participate, six patients withdrew after consent and two patients were excluded owing to protocol breaches. Consequently, a total of 120 patients were analyzed and completed the postoperative follow-up in the study (Fig. 1).

There were no significant differences in demographic and surgical characteristics between the two groups (*P* > 0.05) (Table 1). Opioid consumption and flurbiprofen-axetil consumption, including PCIA press times were significantly lower in Group E than in Group C (*P* < 0.05) (Table 2).

**Table 1:** Basic data of patients

Categury	Groups		*P*-value
	**Ropivacaine (*n* = 60)**	**Ropivacaine + Dex (*n* = 60)**	
Age (years)	56.40 ± 7.87	54.77 ± 8.61	0.280
Gender (male/female)	30/30	31/29	0.855
Weight (Kg)	63.78 ± 6.34	64.35 ± 6.12	0.619
Height (cm)	165.70 ± 7.86	165.80 ± 7.38	0.943
ASA status			0.572
I	24 (40%)	21 (35%)	
II	36 (60%)	39 (65%)	
Anesthesia time(min)	152.60 ± 16.47	152.55 ± 15.37	0.986
Surgery time (min)	141.48 ± 17.13	141.70 ± 15.71	0.943
PCA bolus	3 (3–4)	2.5 (2–3)	0.023*

Data were presented as mean ± standard deviations or median (interquartile range). Compared with the control group, *P < 0.05

**Table 2:** Opioid and Flurbiprofen axetil consumption

Drug	Groups		*P*-value
	**Ropivacaine (*n* = 60)**	**Ropivacaine + Dex (*n* = 60)**	
Sufentanil (μg)	31.89 ± 3.17	31.21 ± 2.97	0.226
Remifentanil (mg)	1.46 ± 0.20	1.38 ± 0.21	0.039*
Flurbiprofen axetil (mg)	89.83 ± 43.94	66.67 ± 39.77	0.016*

All quantitative data were presented as mean ± standard deviations. Compared with the control group, *P < 0.05

**Fig. 2:**
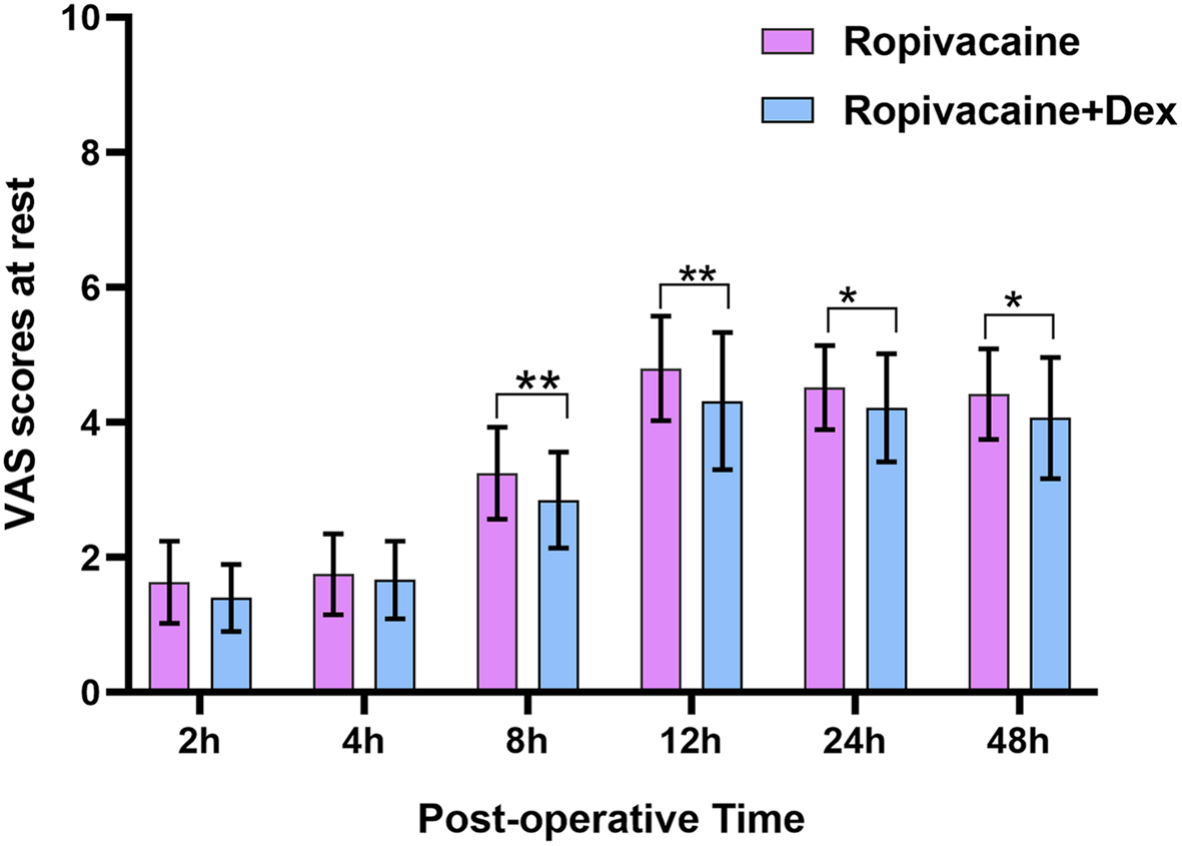
Comparison of VAS at rest between the groups. VAS values were significantly lower in Ropivacaine + Dex group than in Ropivacaine group at 8, 12, 24, and 48 h postoperatively (*P < 0.05), but the VAS values were similar between the groups during the postoperative 2–4 h (*P* > 0.05)

**Fig. 3:**
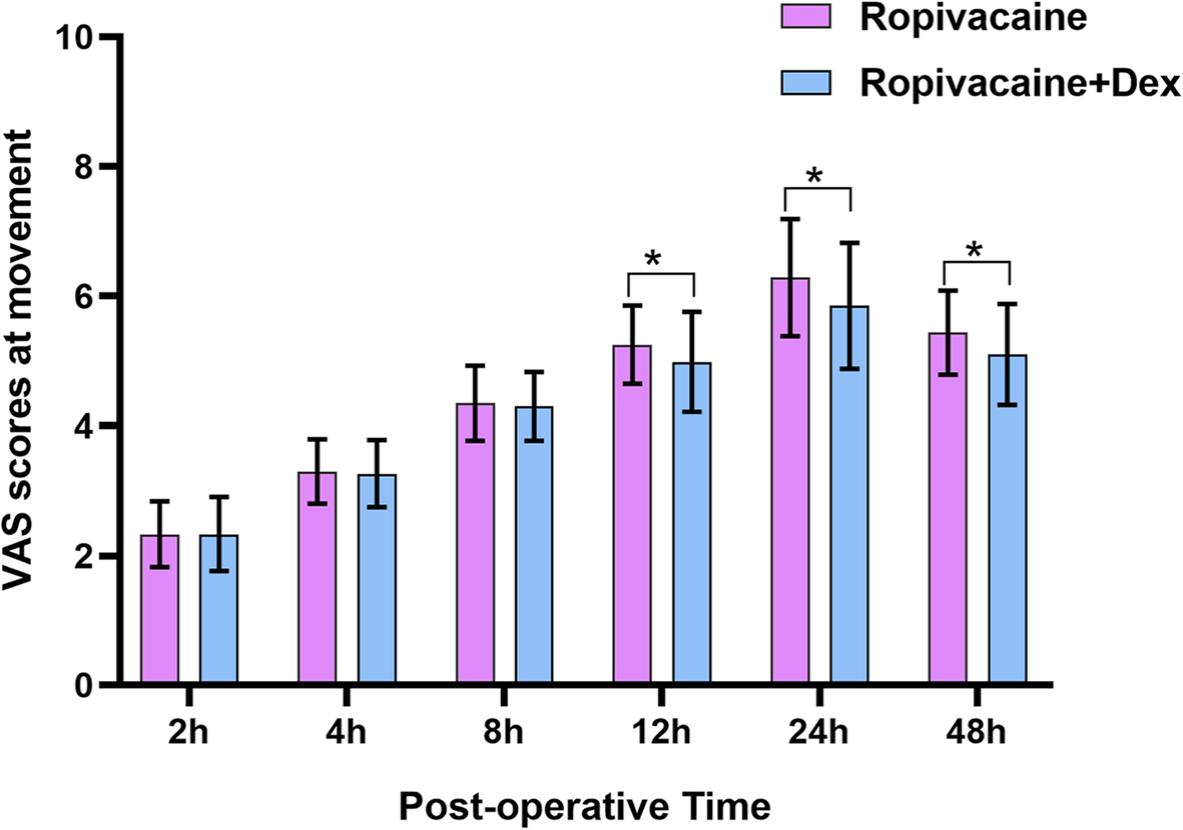
Comparison of VAS at movement between the groups. VAS values were significantly lower in Ropivacaine + Dex group than in Ropivacaine group at 12, 24, and 48 h postoperatively (*P < 0.05), but the VAS values were similar between the groups during the postoperative 2–8 h (*P* > 0.05)

Postoperative VAS pain scores were assessed at rest and during movement. Briefly, the scores at rest and during movement were significantly lower in Group E than in Group C at 12, 24 and 48 h postoperatively (*P* < 0.05) (Figs. 2 and 3). However, at 2,4 and 8 h postoperatively, the scores during movement were similar between the two groups (*P* = 0.087, *P* = 0.092, *P* = 0.109, respectively) (Fig. 3), but at rest the scores were similar between the two groups only at 2 and 4 h postoperatively (*P* = 0.075 and *P* = 0.0.89, respectively), (Fig. 3).

Additionally, the QoR-15 scores after surgery at POD1 and 2 were significantly higher in Group E than in Group C (median 107, IQR 103–113 vs. median 103.5, IQR 104.25–119; *P* = 0.016 and median 116, IQR 111.25–120 vs. median 112, IQR 102–116.75; *P* = 0. 0.021). However, no significant difference was determined between the two groups in QoR-15 scores at POD7 (*P* = 0.190) (Table 3).

**Table 3:** QoR-15 Scores after surgery

Postoperative day	Groups		*P*-value
	**Ropivacaine (*n* = 60)**	**Ropivacaine + Dex (*n* = 60)**	
1	103.50 (104.25–119.00)	107.00 (103.00–113.00)	0.016*
2	112.00 (102.00–116.75)	116.00 (111.25–120.00)	0.021*
7	128.00 (122.00–133.00)	129.50 (125.25–135.00)	0.190

Data were presented as median (interquartile range). Compared with the control group, *P < 0.05

As detailed in Table 4, the patients in Group E performed better in the pain intensity domain of the APS-POQ-R questionnaire on the first postoperative day compared with Group C (worst pain, median 6, IQR 6–7 vs. median 6, IQR 6–7; *P* = 0.035; average pain, median 4, IQR 4–5 vs. median 5, IQR 4–5; *P* = 0.021). In addition, the percentage of time that patients experienced severe pain in the first 24 h was significantly different (median 20, IQR 10–30 vs. median 20, IQR 10–20; *P* = 0.015). The percentage of pain relief received from pain treatments was higher in Group E than that in Group C (median 70, IQR 70–80 vs. median 70, IQR 60–70; *P* = 0.042). In addition, the degree of pain interfering with or preventing activities and sleep was also different between the two groups (*P* < *0.*05). In addition, the patient satisfaction scores were higher in Group E than in Group C (median 8, IQR 8–9, vs. median 8, IQR 7–9; *P* = 0.011). However, no significant differences in uncomfortable feelings and side effects were observed (*P* > 0.05) (Table 4).

**Table 4:** APS-POQ-R questionnaire results 24 h after ESPB

	**Groups**		***P*-value**
	**Ropivacaine (*n* = 60)**	**Ropivacaine + Dex (*n* = 60)**	
**Pain intensity**			
least pain	2.00 (1.00–2.00)	2.00 (1.00–2.00)	0.665
worst pain	7.00 (6.00–7.00)	6.00 (6.00–7.00)	0.034*
average pain	5.00 (4.00–5.00)	4.00 (4.00–5.00)	0.021*
% of time in severe pain in the first 24 h	20.00 (10.00–30.00)	20.00 (10.00–20.00)	0.015*
% of pain relief in the first 24 h	70.00 (60.00–70.00)	70.00 (70.00–80.00)	0.042*
Pain interfered or prevented activities			
in bed	6.00 (5.00–6.75)	5.00 (5.00–6.00)	0.042*
out of bed	7.00 (7.00–8.00)	7.00 (6.00–7.00)	0.061
Pain interfered or prevented sleep			
falling asleep	3.00 (2.00–4.00)	3.00 (2.00–3.00)	0.026*
staying asleep	3.00 (3.00–4.00)	3.00 (2.00–3.00)	0.020*
Pain caused you to feel			
anxious	2.00 (1.00–3.00)	1.00 (0.00–2.00)	0.096
depressed	0.00 (0.00–1.00)	0.00 (0.00–1.00)	0.641
Side effects			
nausea	3.00 (2.00–4.00)	3.00 (2.00–4.00)	0.657
drowsiness	0.00 (0.00–1.00)	0.00 (0.00–1.00)	0.832
Patient perception of their pain management			
participation in pain management	8.00 (8.00–9.00)	9.00 (8.00–10.00)	0.553
satisfaction with pain management	8.00 (7.00–9.00)	8.00 (8.00–9.00)	0.011*

Data were presented as median (interquartile range). Compared with the control group, *P < 0.05

## Discussion

Lumbar spine surgery is frequently performed to relieve pain and provide functional improvement in patients with spinal stenosis and degenerative disc disease. During surgery, mechanical and thermal trauma can cause muscle ischemia and damage to nerves innervating the paraspinal muscles. Therefore, it is often characterized by severe and diffuse pain in the postoperative period [22, 23]. ERAS protocols primarily involve the use of regional anesthesia techniques to minimize opioid analgesics whenever possible. Recently, ESPB as a new trunk fascia block technique was proposed in 2016 [24]. However, few clinical studies have focused on ESPB in lumbar surgery. Moreover, there are differences in the mechanism and effect of block in different parts of the erector spinal muscle [25]. Some believe that ESPB can block the posterior root of the spinal nerve and produce part of the paraspinal block effect with diffusion of the drug solution [26]. Other researchers have found that local anesthetic spread well, was volume dependent, and extended into the neural foramina and epidural space normally. At the same time, local anesthetic can show significantly more epidural spread when the lamina and ligaments are compromised [27]. Based on these studies, it seems likely that ESPB primarily anesthetizes the dorsal rami of the spinal nerves, which innervate the paraspinal muscles and posterior bony elements of the spine. Although indignant peripheral nerve catheters can significantly prolong the analgesic time, they are neither ideal nor feasible for patients in hospitals. Therefore, a method is still needed to expand the analgesic effect of postoperative single-injection nerve block.

To the best of our knowledge, clinical trials have previously shown that various adjuvants for local anesthetics have significant effects, but there have been few studies of the use of dexmedetomidine as a local anesthetic adjuvant in ESPB, and none have satisfactorily assessed the quality of postoperative recovery [28]. In a previous study by Gao et al. [29], the authors found that the block time of ESPB could be prolonged by approximately 120% by adding dexmedetomidine (1 µg/kg) to 0.5% ropivacaine. In addition, a recent study also revealed that 1 μg/kg of dexmedetomidine combined with 0.33% ropivacaine ESPB could better provide postoperative analgesia than without dexmedetomidine, thus improving postoperative analgesia and comfort levels [20]. In this study, we found that adding of 1 of µg/kg dexmedetomidine to 0.375% ropivacaine had a better analgesic effect at 12, 24 and 48 h after surgery, while there was no significant difference in the analgesic effect between the two groups at 2 and 4 h after surgery. The main reason was that ropivacaine nerve block alone had difficulty maintaining a good anesthesia effect after 6–8 h [15].

During the course of this study, adding 1 µg/kg of dexmedetomidine to ESPB did not cause significant fluctuations in MAP or HR. In addition, there was no significant difference in the incidence of nausea, vomiting, dizziness or other adverse reactions between the two groups. However, the results did not indicate that any dose of dexmedetomidine was safe for perineural injection. In a previous study, the researchers found that adding 100 µg of dexmedetomidine to local anesthetic resulted in a significant decrease in blood pressure and HR during the first 2 h after surgery [30]. Furthermore, a meta-analysis showed that the likelihood of intraoperative bradycardia was significantly increased when dexmedetomidine was injected perineurally at a dose > 50 µg [18]. More importantly, the most common adverse reactions to dexmedetomidine were bradycardia and hypotension, which occurred mainly in elderly individuals [15]. Therefore, no significant bradycardia or hypotension was observed in our study, mainly because the dose of dexmedetomidine was not high, and the patients involved in the study were not very old.

Furthermore, we chose the QoR-15 score and APS-POQ-R questionnaire as the secondary outcomes, mainly because the Standardized Endpoints in Perioperative Medicine initiative stated that one or more of six recommended endpoints should be considered in clinical trials evaluating patient comfort after surgery [31]. One of them was the QoR-15 score, which was developed from the longer QoR-40, fulfilling the requirements for outcome measurement instruments in clinical trials, and it was the first measurement instrument of postoperative quality of recovery to undergo a systematic review according to the COSMIN checklist [32]. In our study, we found that ESPB with dexmedetomidine and ropivacaine resulted in increased 4 -point global QoR-15 scores at POD1. Therefore, our results indicated that dexmedetomidine with ropivacaine for ESPB led to significantly better postoperative health status of patients after posterior lumbar spine surgery. Additionally, the APS-POQ-R as a valid measure of the quality of postoperative pain management used internationally for patients was applied in this study, providing a good reference value for us. We observed that perineural dexmedetomidine was more effective in alleviating postoperative pain intensity and improving patients' sleep than a single injection of ropivacaine after posterior lumbar spine surgery. Furthermore, the satisfaction score for pain management in the intervention group was higher than that in the control group. Most likely, the intervention group had prolonged pain relief time, lower pain scores, better sleep quality, and fewer postoperative adverse reactions, which were all factors affecting patients' satisfaction with pain management.

Our study demonstrated that a single injection of ropivacaine provides sensory block for 6–8 h, and postoperative pain often increases opioid consumption and causes opioid-induced side effects, including respiratory depression, nausea and vomiting [33, 34]. However, a single injection ESPB of dexmedetomidine with ropivacaine could extend the sensory block to 18–24 h and provided comfortable analgesia and sleep on the first postoperative night, facilitating early patient activity and reducing the risk of pulmonary complications, thus reduced the length of hospital stay. Our results were similar to multiple studies suggesting that perineural application of dexmedetomidine prolonged the block time and reduced the number of PCA compressions and the need for postoperative rescue analgesia [35, 36]. Therefore, all of the findings suggested that dexmedetomidine combined with ropivacaine for ESPB, as a part of the multimodal analgesia approach, could be a more effective intervention for enhanced recovery after posterior lumbar spine surgery.

However, the main mechanism associated with the action of dexmedetomidine in improving blockade efficacy remains unclear. Previous studies have included the following three hypotheses. First, dexmedetomidine causes vasoconstriction which delays absorption of the local anesthetic and prolongs the effect of local anesthetics [37, 38]. Second, dexmedetomidine blocks hyperpolarization-activated cationic currents and reduces acute local anesthetic-induced perineural inflammation without causing nerve damage [39]. Finally, dexmedetomidine itself has analgesic effects and analgesic retention properties, and peripheral α_2A_-ARs are the mechanism of dexmedetomidine in the treatment of peripheral nerve block pain [40].

Our study had several limitations. First, the assessment methods of pain levels and sensory blockade were limited to subjective sensation of pain and cold. Second, the study was a small, randomized, double-blind trial closely integrated with clinical application. Further large-scale trials are needed to evaluate the clinical efficacy of dexmedetomidine as a local anesthetic adjuvant. Finally, there have been few studies of the mechanism of peripheral dexmedetomidine in ESPB. Therefore, it is necessary to conduct in-depth studies of the preclinical toxicity and clinical application of dexmedetomidine as a local anesthetic adjuvant to clarify its mechanism of action and safe optimal doses to provide a maximum benefit while minimizing side effects in ESPB.

## Conclusion

Dexmedetomidine, as an adjunct to ropivacaine used in ESPB, can prolong sensory block time, effectively control postoperative acute pain, reduce the need for remedial analgesia, and improve postoperative recovery quality and patient satisfaction. Because of the safety and efficacy of dexmedetomidine, we recommend its widespread use in peripheral nerve block.

## Data Availability

The datasets used and/or analysed during the current study are available from the corresponding author on reasonable request.
